# The novel VEGF receptor 2 inhibitor YLL545 inhibits angiogenesis and growth in breast cancer

**DOI:** 10.18632/oncotarget.9392

**Published:** 2016-05-17

**Authors:** Jianbo Zhang, Chen Liu, Wen Shi, Lingling Yang, Quansheng Zhang, Jianlin Cui, Yangwu Fang, Yuhao Li, Guosheng Ren, Shuang Yang, Rong Xiang

**Affiliations:** ^1^ Chongqing Key Laboratory of Molecular Oncology and Epigenetics, The First Affiliated Hospital of Chongqing Medical University, Chongqing, China; ^2^ Tianjin Key Laboratory of Tumor Microenvironment and Neurovascular Regulation, Medical College of Nankai University, Tianjin, China; ^3^ School of Food and Bioengineering, Xihua University, Sichuan, China; ^4^ Tianjin Key Laboratory of Organ Transplantation, Tianjin First Center Hospital, Tianjin, China

**Keywords:** VEGFR2 inhibitor, angiogenesis, breast cancer, tumor gowth

## Abstract

Their antiangiogenic effects make vascular endothelial growth factor receptor 2 (VEGFR2) inhibitors useful for cancer treatment. However, most of these drugs have unexpected adverse side effects. Here, we show that the novel VEGFR2 inhibitor YLL545 suppressed tumor angiogenesis and growth in triple-negative breast cancer without adverse effects. YLL545 treatment also markedly inhibited proliferation, migration, invasion, and tube formation by human umbilical vascular endothelial cells (HUVECs) *in vitro*. These effects of YLL545 were equal to or greater than those seen with sorafenib. In addition, YLL545 inhibited VEGF-induced phosphorylation of VEGFR2 and activation of downstream signaling regulators, such as phospho-STAT3 and phospho-ERK1/2, in HUVECs. Embryonic angiogenesis assays in zebrafish and Matrigel plug assays in mice demonstrated that YLL545 inhibits angiogenesis *in vivo*. YLL545 also inhibited proliferation and induced apoptosis in MDA-MB-231 breast cancer cells both *in vitro* and *in vivo*, and 50 mg/kg/d YLL545 inhibited human tumor xenograft growth by more than 50% in BALB/c nude mice. These observations suggest YLL545 is a potentially useful anticancer drug candidate.

## INTRODUCTION

Angiogenesis is characterized by the formation of new, irregular blood vessels from a preexisting vascular network and is one of the hallmarks of cancer. This process involves the coordination of many bioactive molecules and is required for the growth, survival, and metastasis of most solid tumors, including breast cancer [1, 2]. Triple-negative breast cancer is an aggressive subtype of breast cancer for which treatment options are limited when chemotherapy is unsuccessful [3, 4]. Therapies that block angiogenesis may help inhibit tumor growth and metastasis. Compared to chemotherapy, which is hindered by the ability of cancer cells to mutate rapidly and acquire drug resistance, antiangiogenic therapy may have many advantages [5, 6].

Of all the known angiogenic molecules, vascular endothelial growth factor (VEGF) is the best-characterized modulator of angiogenesis and metastatic growth in human carcinogenesis [7, 8]. VEGF exerts its biological effects by binding to and activating its receptors (VEGFRs). In endothelial cells, VEGFR2 is the major effector of VEGF-stimulated cell survival and vascular permeability during angiogenesis [9, 10]. Activation of VEGFR2 leads to phosphorylation of specific downstream signal transduction mediators, such as mitogen-activated protein kinase (MAPK), signal transducer and activator of transcription 3 (STAT3), and mechanistic target of rapamycin (mTOR) [11–13]. Therefore, VEGFR2 is an important target for antiangiogenic cancer therapies. A number of small molecule VEGFR2 inhibitors have been reported, and some have been approved by the Food and Drug Administration, including sorafenib, sunitinib, and vandetanib [14–16]. Moreover, novel small molecule VEGFR2 inhibitors, such as isoliquiritigenin and NBM-T-BMX-OS0118, are currently under clinical and preclinical evaluation [17, 18]. However, adverse effects such as bleeding complications [19] have been observed in patients treated with these drugs, indicating that safer VEGFR2 inhibitors are still needed.

In an effort to discover more potent VEGFR2 inhibitors that specifically block VEGF/VEGFR2 signaling with little or no toxicity, we used rational medicinal chemistry methods to design a series of inhibitors derived from sorafenib. Here, we examined the ability of one of these novel small molecules, YLL545, to inhibit VEGFR2. Our findings suggest YLL545 is a well-tolerated novel compound that potently inhibits VEGFR2 and may suppress tumorigenesis in breast cancer.

## RESULTS

### Design, synthesis and evaluation of YLL545

**Figure 1:**
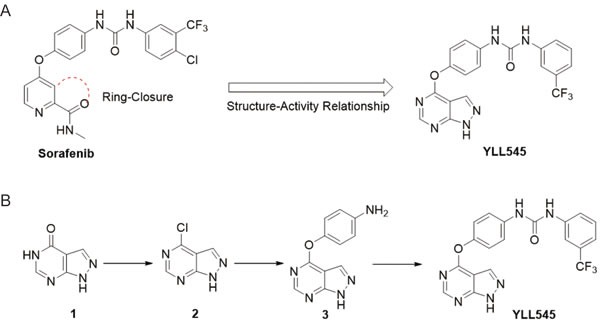
Design, synthesis and evaluation of YLL545 **A.** Schematic showing the design of compound YLL545. **B.** The synthesis route of compound YLL545.

We used rational medicinal chemistry methods, such as ring-closure and structure-activity relationship, to generate potent antiangiogenic agents from sorafenib. This led to the discovery of 1-(4-((1*H*-pyrazolo[3,4-*d*]pyrimidin-4-yl)oxy)phenyl)-3-(3-(trifluoromethyl)phenyl)urea, termed YLL545 (Figure 1A). YLL545 was synthesized from commercially available 1*H*-pyrazolo[3,4-*d*]-pyrimidin-4(5*H*)-one (1) using a chloro reaction with phosphorus oxychloride, a nuclear substitution reaction and a condensation reaction. As shown in Figure 1B, synthesis was performed according to a previously described method [20]. YLL545 was obtained at 46% yield and 98% HPLC purity. ^1^H NMR (400 MHz, DMSO-d_6_): δ 14.14 (s, 1H), 9.10 (s, 1H), 8.93 (s, 1H), 8.51 (s, 1H), 8.04 (s, 2H), 7.61-7.51 (m, 4H), 7.32 (d, *J* = 7.6 Hz, 1H), 7.26 (d, *J* = 8.8 Hz, 2H). ^13^C NMR (100 MHz, DMSO-d6): δ 163.2, 156.7, 154.9, 152.6, 146.6, 140.5, 137.2, 131.8, 129.9, 122.3, 121.8, 119.7, 101.3. HRMS: m/z calcd for C_19_H_13_F_3_N_6_O_2_ [M + H]^+^ 415.1052, found 415.1062.

### YLL545 inhibits HUVEC proliferation, migration, invasion, and tube formation

**Figure 2:**
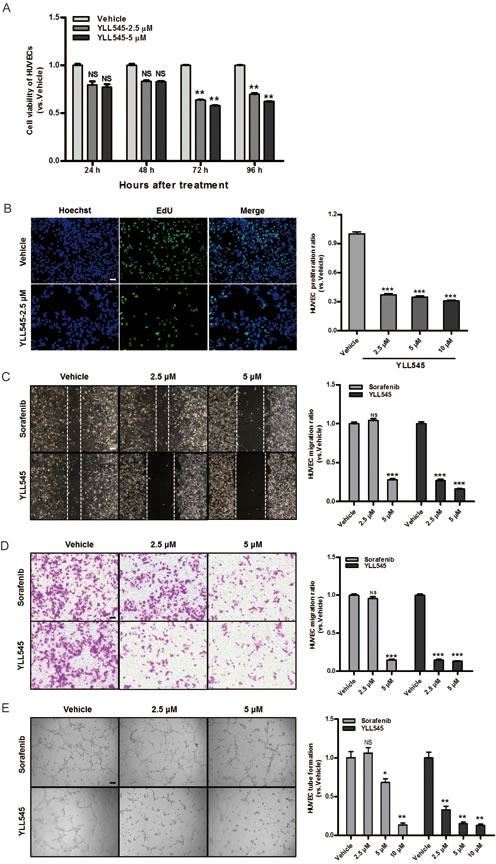
YLL545 inhibits HUVEC proliferation, migration, invasion, and tube formation **A.** Cell viability assays were conducted using HUVECs treated with different concentrations of YLL545. At the indicated time points, cell growth inhibition was determined. **P < 0.01 *vs* respective control in a one-way ANOVA followed by Tukey's HSD test. **B.** EdU proliferation assays were conducted using HUVECs treated with different concentrations of YLL545 for 72 h. ***P < 0.001 *vs* respective control in Student's *t*-test. Scale bars, 50 μm. **C.** HUVECs were wounded using pipette tip and then treated with different concentrations of YLL545 or sorafenib for 24 h. Migrated distances were quantified by manual counting and expressed as a percentage of vehicle-treated cell migaration. ***P < 0.001 *vs* respective control in Student's *t*-test. Scale bars, 100 μm. **D.** HUVECs were seeded in the top chamber and treated with different concentrations of YLL545 or sorafenib. After 16 h, HUVECs that migrated through the membrane were stained and quantified. ***P < 0.001 *vs* respective control in Student's *t*-test. Scale bars, 100 μm. **E.** HUVECs were seeded on a Matrigel layer and treated with different concentrations of YLL545 or sorafenib. After 6 h, tubular structures were manually counted. *P < 0.05 and **P < 0.01 *vs* respective control in Student's *t*-test. Scale bars, 100 μm.

To assess its antiangiogenic activity *in vitro*, we examined the effect of YLL545 on VEGF-induced proliferation in HUVECs using CCK-8 assays. YLL545 inhibited VEGF-induced HUVEC proliferation with an IC_50_ of 5.884 μM (Figure 2A and S1). Moreover, EdU incorporation assays demonstrated that the percentage of proliferating HUVECs decreased after treatment with 2.5 μM YLL545 (Figure 2B). Given that endothelial cell migration and invasion are essential for angiogenesis, we carried out wound healing assays to investigate the effect of YLL545 on HUVEC migration. As shown in Figure 2C, 2.5 μM YLL545 strongly inhibited HUVEC migration; 5 μM sorafenib inhibited cell migration to a similar degree. Transwell invasion assays further revealed that YLL545 dose-dependently inhibited invasive ability in HUVECs compared to sorafenib-treated cells (Figure 2D).

Although angiogenesis is a complex process involving several types of cells, tube formation in endothelial cells is one of the key steps. HUVECs were grown on Matrigel and cultured in different concentrations of YLL545 and sorafenib, and their ability to form capillary tubes was examined. As shown in Figure 2E, treatment with YLL545 dose-dependently inhibited tube formation in HUVECs. HUVECs grown in 2.5 μM YLL545 formed approximately 67% fewer capillary tubes than those grown in vehicle control; 2.5 μM sorafenib -inhibited tube formation to a lesser degree. Collectively, these results indicate that YLL545 had antiangiogenic effects on HUVEC proliferation, migration, invasion, and tube formation, and that these effects were either similar to or stronger than those of sorafenib.

### YLL545 functions *via* VEGFR2-dependent and -independent pathways

**Figure 3:**
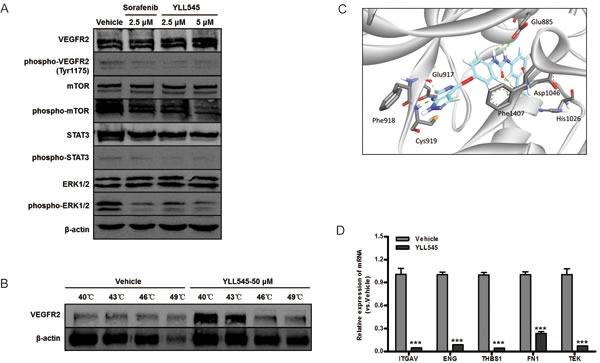
YLL545 functions *via* VEGFR2-dependent and independent pathways **A.** HUVECs were treated with different concentrations of YLL545 and vehicle control for 72 h. The expression and phosphorylation of VEGFR2, mTOR, STAT3, and ERK1/2 were measured by immunoblotting and normalized to levels of β-actin. **B.** Cellular thermal shift assays were conducted using HUVECs treated with 50 μM YLL545 or vehicle control. The expression of VEGFR2 was measured by immunoblotting and normalized to levels of β-actin. **C.** Molecular docking showed the binding mode of YLL545 with the inactive conformation of VEGFR2. **D.** HUVECs were treated with 5 μM YLL545 or vehicle for 24 h. The expression of ITGAV, ENG, THBS1, FN1, and TEK expression were examined by quantitative PCR. Expression levels were normalized to GAPDH expression. ***P < 0.001 *vs* respective control in Student's *t*-test.

To determine whether YLL545 inhibited VEGFR2 and downstream signaling, we screened essential kinases involved in the VEGFR2 signaling pathway. As shown in Figure 3A, treatment with 2.5 μM YLL545 suppressed VEGF-induced phosphorylation of VEGFR2, mTOR, STAT3, and ERK1/2. Given that VEGFR2 might be stabilized by its physical engagement with YLL545, we next examined their interaction in intact HUVECs. The results of a cellular thermal shift assay showed that YLL545 efficiently stabilized VEGFR2 (Figure 3B), demonstrating that YLL545 exerts its antiangiogenic effects by directly targeting VEGFR2 and antagonizing VEGFR2-mediated signaling cascades. Furthermore, we used molecular docking to analyze the binding mode of YLL545 with the inactive conformation of VEGFR2. As shown in Figure 3C, YLL545 formed a hydrogen bond with Cys919 in the VEGFR2 linker region. The urea moiety of YLL545 formed very tight hydrogen-bonding interactions with Glu885 and Asp1046 in the DFG loop. There were also some pi-pi interactions between YLL545 and Phe1407 and hydrophobic interactions with the Ile898, Val899, Ile892, and Val899 residues in the allosteric pocket. These results indicate that YLL545 has a similar binding mode to that of sorafenib [21], demonstrating the effectiveness of our ring closure design strategy.

We then examined differences in mRNA levels of selected angiogenesis signaling molecules using the RT^2^ Profiler PCR Array by comparing YLL545-treated HUVECs with vehicle-treated control. Interestingly, YLL545 altered the expression of several VEGFR2-independent regulators (more than ± 10-fold), including ITGAV, ENG, THBS1, FN1, and TEK were observed (Table 1). These results were confirmed by quantitative PCR, as shown in Figure 3D, and indicate that YLL545 may regulate endothelial angiogenesis *via* both VEGFR2-dependent and -independent pathways.

**Table 1:** Genes regulated by YLL545

GeneBankTM accession no.	Symbol	Description	Fold change (YLL545 *vs*. Vehicle)	*P* value
NM_001145000	ITGAV	Integrin, alpha V	−28.5049	<0.0001
NM_001114753	ENG	Endoglin	−16.2973	<0.0001
NM_003246	THBS1	Thrombospondin 1	−12.5946	<0.0001
NM_212482	FN1	Fibronectin 1	−10.5406	<0.0001
NM_000459	TEK	TEK tyrosine kinase, endothelial	−10.1189	<0.0001

### YLL545 inhibits angiogenesis *in vivo*

**Figure 4:**
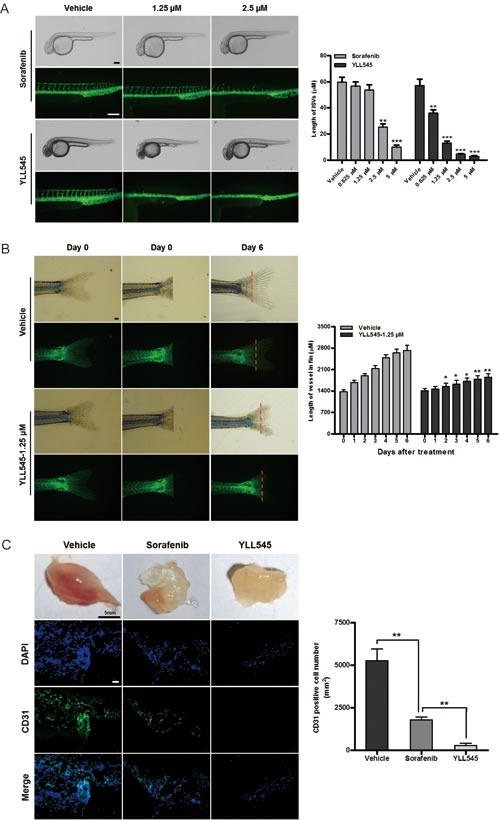
YLL545 inhibits angiogenesis *in vivo* **A.** 12-hpf zebrafish embryos were incubated with different concentrations of YLL545 or sorafenib. At 36 hpf, fluorescent assays were performed to visualize the growth of ISVs and DLAVs, which were manually counted. **P < 0.01 and ***P < 0.001 *vs* respective control in Student's *t*-test. Scale bars, 100 μm. **B.** At the indicated time points, fluorescent assays were performed to visualize the growth of caudal vessels, which were manually counted. *P < 0.05 and **P < 0.01 *vs* respective control in Student's *t*-test. Scale bars, 300 μm. C. Matrigel containing 80 mg/kg YLL545 or 80 mg/kg sorafenib was subcutaneously injected into mice, and the plugs were surgically removed after 10 days. Expression of CD31 in plugs from YLL545- or sorafenib-treated mice was examined by immunofluorescent staining. Scale bars, 100 μm.

To evaluate the antiangiogenic activity of YLL545 *in vivo*, we used a Fli-1 promoter enhanced green fluorescent protein (EGFP) transgenic zebrafish model. Twelve hours post fertilization (hpf) zebrafish embryos were incubated overnight with different concentrations of YLL545 or sorafenib. As shown in Figure 4A, 0.625 to 1.25 μM YLL545 reduced the formation of dorsal longitudinal anastomotic vessels (DLAVs) and intersegmental vessels (ISVs) compared to sorafenib treatment; the formation of major cranial vessels was not inhibited. Treatment with 2.5 μM YLL545 resulted in a complete loss of DLAVs and ISVs. YLL545 similarly inhibited caudal vessel formation (Figure 4B), indicating that YLL545 effectively inhibits angiogenesis *in vivo* in zebrafish.

Next, we conducted Matrigel plug assays in a mouse model of angiogenesis. As shown in Figure 4C, new blood vessels in Matrigel plugs were inhibited in mice treated with 80 mg/kg YLL545 compared to the vehicle-treated group. Immunofluorescent staining of an anti-CD31 antibody (Ab) further revealed that YLL545 treatment reduced the number of endothelial cells embedded in the plug sections by 95%. Sorafenib inhibited the formation of new blood vessels in Matrigel plugs to a lesser degree. These data reveal that YLL545 blocks new blood vessel formation *in vivo*.

### YLL545 inhibits tumor cell growth *in vitro*

**Figure 5:**
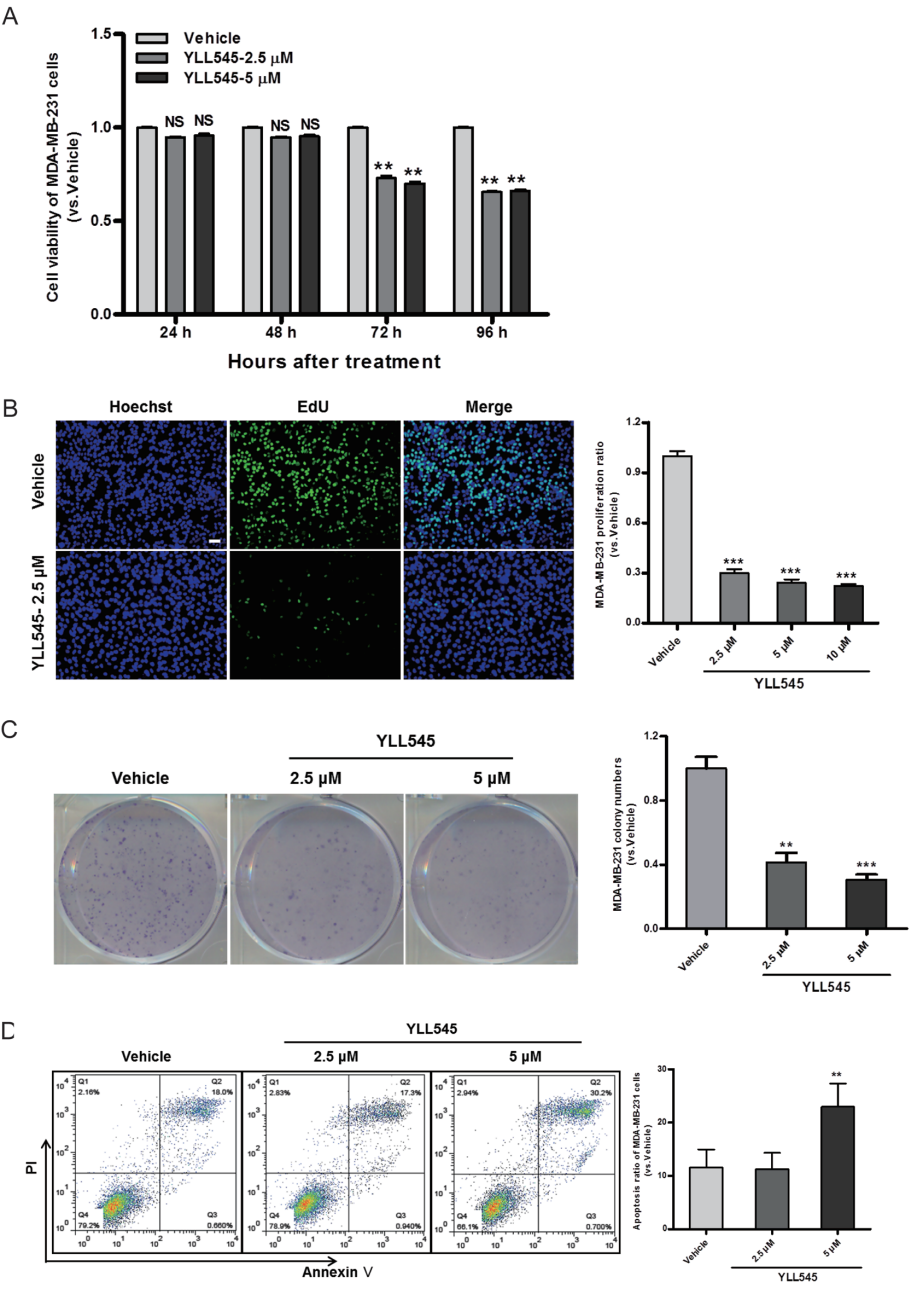
YLL545 inhibits tumor cell growth *in vitro* **A.** Cell viability assays were conducted using MDA-MB-231 cells treated with different concentrations of YLL545. At the indicated time points, cell growth inhibition was determined. **P < 0.01 *vs* respective control in a one-way ANOVA followed by Tukey's HSD test. **B.** Colony formation assays were conducted using MDA-MB-231 cells treated with different concentrations of YLL545 for 7 days. **P < 0.01 and ***P < 0.001 *vs* respective control in Student's *t*-test. **C.** EdU proliferation assays were conducted using MDA-MB-231 cells treated with different concentrations of YLL545 for 72 h. ***P < 0.001 *vs* respective control in Student's *t*-test. Scale bars, 50 μm. **D.** MDA-MB-231 cells were treated with different concentrations of YLL545 for 72 h, and annexin V/PI staining was analyzed using flow cytometry. The proportions of early apoptotic cells (annexin V-positive) and late apoptotic cells (PI-positive) are shown. **P < 0.01 *vs* respective control in Student's *t*-test.

Because VEGF/VEGFR2 are also highly expressed in triple-negative breast cancer cells [22, 23], we explored the potential role of YLL545 in the regulation of tumor growth. To do this, we measured cell viability in MDA-MB-231 cells treated with different concentrations of YLL545 using CCK-8 assays. As shown in Figure 5A, YLL545 inhibited MDA-MB-231 cell viability in a dose-dependent manner, with an IC_50_ value of 13.34 μM (Figure S2). Colony formation assays confirmed that 2.5 μM YLL545 decreased colony formation in MDA-MB-231 cells (Figure 5B). Inhibition of cell proliferation is a possible approach for cancer treatment; we therefore performed an EdU incorporation assay to investigate the antiproliferative effect of YLL545. As shown in Figure 5C, 2.5 μM YLL545 decreased the percentage of proliferating MDA-MB-231 cells by 70%. Annexin V/propidium iodide (PI) staining was used to examine YLL545-induced cell apoptosis. Treatment with 5 μM YLL545 markedly increased apoptosis in MDA-MB-231 cells (Figure 5D). These observations collectively suggest YLL545 strongly inhibits tumorigenesis *in vitro*.

### YLL545 exerts antitumor activities *in vivo*

**Figure 6:**
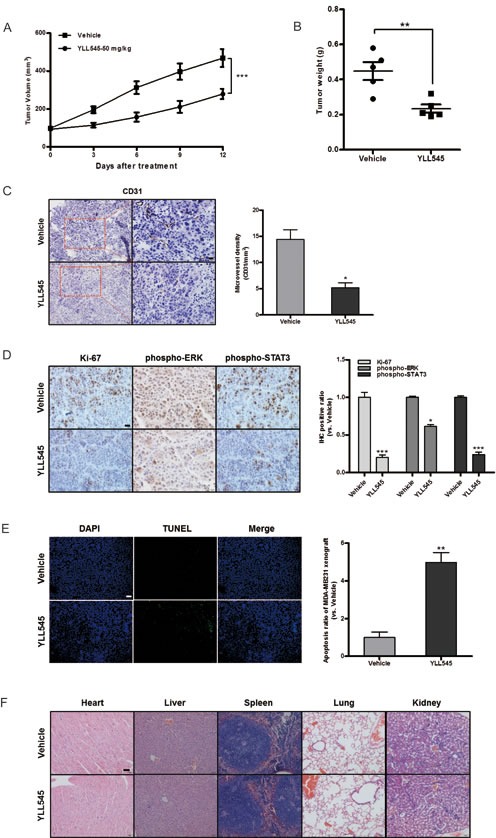
YLL545 exerts antitumor activities *in vivo* **A.** and **B.** A total of 2.5×10^7^ MDA-MB-231 cells were injected into the fat pad of nude mice (*n* = 5). Tumor development was monitored for 14 days. When tumors reached a volume of about 100 mm^3^, the mice were treated with 50 mg/kg/d YLL545 or vehicle for another 12 days. Cross-sectional diameters of tumors from YLL545- and vehicle-treated mice were measured. Approximate tumor volumes (A) and weights (B) were calculated as described in Materials and Methods. **P < 0.01 and ***P < 0.001 *vs* respective control in Student's *t*-test. **C.** Expression of CD31 in breast cancer xenografts was examined by immunohistochemical staining. MVD was determined by CD31 staining. MVD was counted in five random fields in each tumor. *P < 0.05 *vs* respective control in Student's t-test. Scale bars, 20 μm. **D.** Ki67, phospho-STAT3 and phospho-ERK1/2 levels in breast cancer xenografts were examined by immunohistochemical staining. *P < 0.05 and ***P < 0.001 *vs* respective control in Student's *t*-test. Scale bars, 20 μm. **E.** YLL545-induced tumor cell apoptosis was measured by TUNEL staining in YLL545- and vehicle-treated mice. **P < 0.01 *vs* respective control in Student's *t*-test. Scale bars, 100 μm. **F.** YLL545 did not cause obvious pathologic abnormalities in normal tissues. H&E staining of paraffin embedded sections was performed on heart, liver, spleen, lung, and kidney tissues from YLL545- and vehicle-treated mice. Scale bars, 50 μm.

Next, to better assess the effect of YLL545 on tumor growth *in vivo*, a nude mouse xenograft model was established using MDA-MB-231 cells. As shown in Figure 6A and 6B, treatment with 50 mg/kg/d YLL545 resulted in ~50% inhibition of tumor growth compared to vehicle-treated mice. Immunohistochemical staining for anti-CD31 Ab confirmed that tumor microvessel density (MVD) decreased in YLL545-treated mice (Figure 6C). Immunohistochemical staining revealed that Ki67, phospho-ERK1/2, and phospho-STAT3 levels were also reduced in YLL545-treated tumors, indicating decreased cell proliferation and VEGFR2 activity (Figure 6D). Furthermore, we investigated the effect of YLL545 on apoptosis in MDA-MB-231 xenograft tumors using TUNEL staining. As shown in Figure 6E, 50 mg/kg/d YLL545 increased the number of apoptotic cells in tumor tissues, which was consistent with the results from MDA-MB-231 cells *in vitro*. No adverse effects in other gross measures such as skin ulcerations or toxic death, were observed in the YLL545-treated group. Toxic pathological changes in the heart, liver, spleen, lung and kidney were not detected by microscopic examination (Figure 6F). These data indicate that YLL545 both inhibits angiogenesis and induces apoptosis in breast cancer xenografts *in vivo*, and this effect is not attributable to systemic toxicity.

## DISCUSSION

Antiangiogenic therapy is an effective strategy for the treatment of solid tumors [5, 24], and targeting VEGFR2 with small molecule inhibitors of receptor tyrosine kinase has been particularly effective in human cancer treatment [14–16]. In the present study, we investigated a potent new small molecule VEGFR2 inhibitor, YLL545. YLL545 inhibited VEGF-induced VEGFR2 phosphorylation and activation of downstream signaling pathways, such as phospho-STAT3 and phospho-ERK1/2. Functional assays revealed that, YLL545 inhibited proliferation, migration, invasion, and tube formation in HUVECs *in vitro*; the potency of YLL545 was either comparable to or higher than sorafenib in these tests. Notably, YLL545 also strongly inhibited tumor growth and angiogenesis in MDA-MB-231 triple-negative breast cancer cells *in vitro* and *in vivo*. YLL545 might therefore be a promising anticancer drug candidate.

VEGFR2 signaling is essential for vascular endothelial cell function [9]. Tyr^1175^ is the major autophosphorylation site within VEGFR2, and its phosphorylation initiates various downstream signaling events. For example, VEGFR2 with phosphorylated Tyr^1175^ activates the MAPK/ERK cascade and endothelial cell proliferation [25]. Meanwhile, VEGF-induced activation of STAT3 also plays a critical role in angiogenesis, and this signaling pathway is a potential target for antiangiogenic tumor therapy in endothelial cells [26–28]. Recent studies show that STAT3 directly activates VEGF and HIF-1α transcription under hypoxic conditions, which results in the initiation of endothelial cell migration and angiogenesis [29]. Angiogenesis is a complex process with multiple steps [7, 8]. All known angiogenesis inhibitors block one or more steps of these steps by targeting endothelial cells [26]. Here, by directly blocking VEGFR2 phosphorylation, YLL545 suppressed STAT3 and ERK1/2 signaling and ultimately inhibited various processes in endothelial cells *in vitro*, including proliferation, migration, invasion, and tube formation. Moreover, a cellular thermal shift assay confirmed that YLL545 binds to VEGFR2, demonstrating that YLL545 exerts its antiangiogenic function by directly targeting VEGFR2 and antagonizing downstream signaling cascades. In addition to blocking VEGFR2, YLL545 altered the expression of other molecules involved in tumor angiogenesis, including ITGAV [30], ENG [31, 32], THBS1 [33, 34], FN1 [35–37], and TEK [38, 39]. However, these genes are not targets of VEGF/VEGFR2 (Figure S3), indicating that a VEGFR2-independent mechanism might also contribute to the antiangiogenic effects of YLL545.

Because endothelial cell models of migration, invasion, and tube formation lack the biological complexity of vessel systems in vertebrate animals [40], we examined the antiangiogenic effect of YLL545 *in vivo* using zebrafish embryonic angiogenesis and Matrigel plug assay modles. Treatment with 0.625 to 1.25 μM YLL545 inhibited angiogenic formation of DLAVs and ISVs in zebrafish, which corresponds to capillary sprouting in mammals [41]. Importantly, YLL545 had higher or comparable antiangiogenic potency, but strikingly lower cytotoxicity, than sorafenib in zebrafish embryos (Figure S4). Furthermore, subcutaneous transplantation of Matrigel plugs revealed that YLL545 inhibited angiogenic response in mice, confirming that YLL545 blocks the formation of new blood vessels *in vivo*.

In addition to inhibiting endothelial angiogenesis, YLL545 also directly inhibited triple-negative breast cancer cell proliferation and survival. YLL545 inhibited MDA-MB-231 cell proliferation by increasing G1/S cell cycle arrest, as shown in the EdU-incorporation assay. Importantly, YLL545 administration (50 mg/kg/d) inhibited tumor growth by 50% in our BALB/c nude mouse xenograft model. Additionally, treating tumor cells with YLL545 increased the number of apoptotic cells, as visualized by TUNEL staining. As suggested by a previous report [42], it is plausible that YLL545 inhibits angiogenesis in xenograft tumors, which deprives tumor cells of oxygen and nutrients and results in tumor cell apoptosis. Because of these antitumor effects and because it did not induce toxic pathological changes in mice, YLL545 might be a promising novel anticancer candidate drug with limited side effects.

In summary, our studies showed that the novel and well-tolerated small molecule YLL545 inhibited VEGFR2 angiogenesis, and tumorigenesis in triple-negative breast cancer. YLL545 inhibited breast tumor growth and angiogenesis by blocking VEGFR2-mediated downstream signaling pathways, such as STAT3 and ERK1/2. Our research suggests that YLL545 and its derivatives may be potential drug candidates for diseases associated with pathological angiogenesis.

## MATERIALS AND METHODS

### Cell culture

MDA-MB-231 human breast cancer cells were maintained in L15 (Corning) supplemented with 10% fetal bovine serum (FBS) (Biological Industries), penicillin, and streptomycin (Invitrogen). HUVECs were isolated from human umbilical cord, which was provided by Tianjin First Central Hospital, Tianjin, China. HUVECs were maintained in endothelial basal medium-2 (EBM-2) supplemented with a SingleQuots Kit containing VEGF and other growth factors (LONZA). HUVECs at passages 3 to 8 were used for all studies.

### Cell viability assay

Cells growing in 96-well plates were cultured in the presence of different concentrations of YLL545. At the indicated time points, cell viability was assessed using the CCK-8 assay according to the manufacturer's protocols (Dojindo). Six parallel replicates were measured for each sample.

### 5-ethynyl-2′-deoxyuridine (EdU) proliferation assay

Cells growing in 24-well plates were cultured in the presence of different concentrations of YLL545 for 72 h, and then assayed with a Cell-Light™ EdU Apollo^®^488 *In Vitro* Imaging Kit according to the manufacturer's instructions (RiboBio). Images were taken and analyzed using a Confocal FV1000 microscope (Olympus). Percentages of EdU-positive cells were calculated as follows: (EdU-positive cells/Hoechst stained cells) × 100%. At least 200 cells were counted per well.

### Wound healing assay

Cells were allowed to grow to full confluence in 6-well plates and then wounded by scratching with pipette tips, followed by treatment with different concentrations of YLL545 or sorafenib for 24 h. The migrated distance was quantified by manual counting and photographed under a light microscope (Olympus). Migrated distance is expressed as a percentage of the distance observed in vehicle-treated cells.

### Transwell invasion assay

Transwell *in vitro* invasion 24-well chambers (Corning) were used per the manufacturer's instructions. Briefly, the filter was pre-coated with 50 μL Matrigel (BD Biosciences), which was allowed to solidify at 37°C for 1 h. Cells (4.0×10^4^ cells/well for HUVECs, 2.0×10^4^ cells/well for MDA-MB-231) were then placed in the top chambers and treated with different concentrations of YLL545 or sorafenib. Cells were then allowed to migrate for 16 h. Non-migrated cells were scraped with a cotton swab, and migrated cells were fixed with 20% methanol and stained with 0.5% crystal violet. The cells were counted and photographed under a light microscope (Olympus). Cell migration is expressed as a percentage of the migration observed in vehicle-treated cells.

### Tube formation assay

A pre-chilled 48-well plate was coated with 160 μL Matrigel (BD Biosciences), which was allowed to solidify at 37°C for 30 min. Cells (4×10^4^ cells/well) were seeded on the Matrigel and cultured in the presence of different concentrations of YLL545 or sorafenib. After incubation at 37°C for 6 h, 5 randomly chosen fields were counted and photographed under a light microscope (Olympus).

### Colony formation assay

Cells (6×10^2^ cells/well) were seeded in six-well plates and cultured in the presence of different concentrations of YLL545 for 7 days. The cells were fixed with 20% methanol, stained with 0.5% crystal violet and photographed under a light microscope (Olympus).

### Immunoblotting assay

Preparation of total cell extracts and immunoblotting with appropriate antibodies was performed as previously described [43]. The following antibodies (Abs) were used: goat monoclonal Ab against VEGFR2 (# 2479, Cell Signaling Technology) at a dilution of 1:1000, rabbit monoclonal Ab against phospho-VEGFR2 (# 2478, Cell Signaling Technology) at a dilution of 1:1000, rabbit monoclonal Ab against STAT3 (# 14801, Cell Signaling Technology) at a dilution of 1:1000, rabbit monoclonal Ab against phospho-STAT3 (# 4093, Cell Signaling Technology) at a dilution of 1:1000, rabbit monoclonal Ab against ERK1/2 (# 4695, Cell Signaling Technology) at a dilution of 1:1000, rabbit monoclonal Ab against phospho-ERK1/2 (# 14474, Cell Signaling Technology) at a dilution of 1:1000, mouse monoclonal Ab against mTOR (# 4517, Cell Signaling Technology) at a dilution of 1:1000, and rabbit monoclonal Ab against phospho-mTOR (# 5536, Cell Signaling Technology) at a dilution of 1:1000.

### Target engagement assay

The cellular thermal shift assay was performed as previously described [44, 45], with some modifications. Briefly, cells were incubated with 50 μM YLL545 for 3 h, detached with trypsin and collected in TBS containing a complete protease inhibitor cocktail. The cells were then divided into 4 aliquots and heated individually at 40°C, 43°C, 46°C, and 49°C for 3 min. Subsequently, the cell suspensions were freeze-thawed three times using liquid nitrogen. Soluble proteins were separated from the precipitated fraction by centrifugation at 20,000 g for 20 min. The proteins were then detected with an immunoblotting assay using the VEGFR2 Ab at a dilution of 1:1000.

### RT^2^ profiler PCR array

Total RNA was extracted from HUVECs treated with 5 μM YLL545 or vehicle control for 24 h. An RT^2^ Profiler custom PCR array was used to simultaneously examine the mRNA levels of 84 genes closely associated with angiogenesis and 5 housekeeping genes in 96-well plates following the manufacturer's protocol (PAHS-049Z, QIAGEN). Briefly, first-strand cDNAs were synthesized from 1 μg of total RNA using the TaqMan RT reagent kit (QIAGEN). Arrays were performed independently at least 3 times; values were obtained for the threshold cycle (ΔCt) for each gene and normalized using the average of 5 housekeeping genes on the same array (ACTB, B2M, GAPDH, HPRT1, and RPLP0). Changes in expression (ΔΔCt) between YLL545-and vehicle-treated cells were determined using the following formula: ΔΔCt = ΔCt(YLL545) - ΔCt(Vehicle). Fold-change was determined as follows: fold-change = 2^(−ΔΔCt)^. Only genes with a 10-fold or greater change in expression were examined. Negative controls ensured the absence of DNA contamination and defined thresholds for determining absence *versus* presence of expression.

### Drug studies in zebrafish

The transgenic zebrafish embryos (Fli-1: EGFP) used in the experiments were grown and maintained as described previously [46]. 12-hpf zebrafish embryos were treated with different concentrations of YLL545 or sorafenib. At 36 hpf, embryos were anesthetized with 0.01% tricaine, and fluorescent images were captured using a fluorescent microscope (Olympus). Blood vessel formation was quantified by calculating the length of intersegmental vessels (ISVs) and represented as percentage of vehicle control.

### Matrigel plug assay

All experimental procedures involving animals were performed according to institutional ethical guidelines for animal experiments and approved by the Ethics Committee for Animal Use at the Medical College of Nankai University. BALB/c mice (mean body weight: 22.8 ± 0.5 g; n = 4) were given subcutaneous injections of 1000 μL of a 3:1 mixture of growth factor-reduced Matrigel (BD Biosciences) and YLL545 (80 mg/kg) or sorafenib (80 mg/kg). After 10 days, plugs were harvested, imaged, and snap frozen in liquid nitrogen in the presence of optimum cutting temperature (OCT) compound (Tissue-Tek) before sectioning. Frozen Matrigel sections were fixed in cold methanol and immunostained with a rabbit polyclonal Ab against CD31 (ab28364, Abcam).

### Tumor xenograft experiments

MDA-MB-231 cells were collected, suspended in 100 μL of PBS at a concentration of 2.5×10^7^ cells/mL, and injected into the fat pad of female BALB/c nude mice. When tumors reached a volume of approximately 100 mm^3^, mice were randomized into 2 groups (5 mice per group) and YLL545 (50 mg/kg) or vehicle was administered orally once per day. Tumor volumes (*V*) were calculated by measuring the length (*L*) and width (*W*) of the tumor with calipers and using the following formula: *V* = (*L* × *W^2^*) × 0.5. Tumor tissues were also processed and sectioned for histological evaluation.

### Histopathology and immunohistochemistry

Immunohistochemical analysis of paraffin-embedded sections was performed using the Envision Kit (Dako) following the manufacturer's protocols. Sections were boiled in retrieval solutions to expose antigens. We applied 1:100 dilutions of rabbit polyclonal Ab against CD31 (ab28364, Abcam), rabbit monoclonal Ab against Ki-67 (# 9027, Cell Signaling Technology), rabbit monoclonal Ab against phospho-STAT3 (# 9145P, Cell Signaling Technology), rabbit monoclonal Ab against phospho-ERK1/2 (# 4370P, Cell Signaling Technology), and control primary Abs to the sections. Slides were counterstained with hematoxylin, dehydrated, and mounted. Immunostaining was independently evaluated by 2 pathologists. Apoptotic cells in the tumor tissue were detected by staining using an apoptotic cell detection kit (Merck Millipore).

### Statistical analysis

SPSS 17.0 software (SPSS) was used for statistical analysis. The data from all experiments were presented as the means ± SD and represent three independent experiments. One-way analyses of variance (ANOVA) were used to compare means between treatment groups, and Tukey's HSD (honestly significant difference) tests were used to evaluate the statistically significant differences between groups. Where appropriate, Student's *t*-test for unpaired observations was applied. A *p*-value < 0.05 was considered significant.

## SUPPLEMENTARY FIGURES


