# Supplementary material for: The novel VEGF receptor 2 inhibitor YLL545 inhibits angiogenesis and growth in breast cancer
Source: Oncotarget. 2016 May 17;7(27):41067–80. doi: 10.18632/oncotarget.9392 (PMC5173043; doi:10.18632/oncotarget.9392)
Supplement: Supplementary file 1 [file oncotarget-07-41067-s001.pdf]

The novel VEGF receptor 2 inhibitor YLL545 inhibits angiogenesis and growth in breast cancer

Supplementary Material

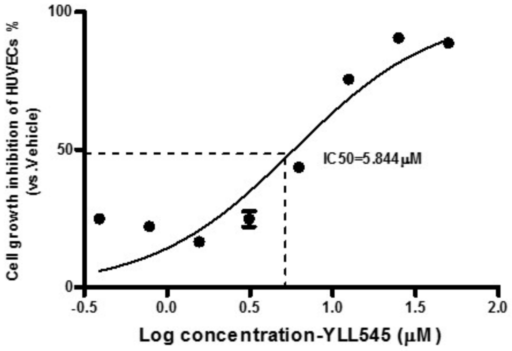

Figure S1. YLL545 inhibits HUVEC cell viability.
The cell viability assays were conducted on HUVECs treated with different concentrations of YLL545. At the indicated time points, cell growth inhibition was determined.

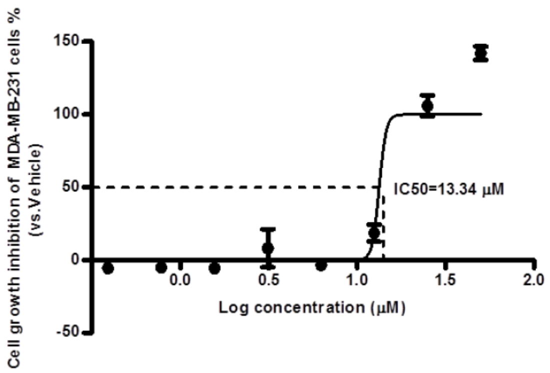

Figure S2. YLL545 inhibits MDA-MB-231 cell viability.

The cell viability assays were conducted on MDA-MB-231 cells treated with different concentrations of YLL545. At the indicated time points, cell growth inhibition was determined.

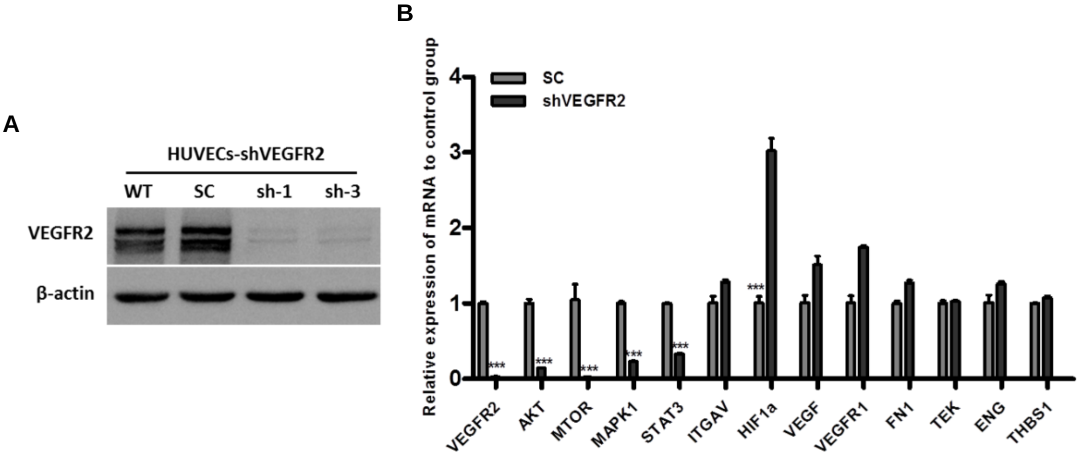

Figure S3. YLL545 functions via VEGFR2-dependent and -independent pathways.

A. HUVECs were stably transfected with specific shRNA targeting VEGFR2 (shVEGFR2) or a scramble control shRNA (SC). The expression of VEGFR2 was verified by immunoblotting and normalized to the levels of β-actin. **B.** The expression of VEGFR2, AKT, mTOR, MAPK1, STAT3, HIF1α, VEGF, VEGFR1, ITGAV, FN1, TEK, ENG, and THBS1 were examined by quantitative PCR in shVEGFR2 or SC cells. GAPDH was used to normalize the individual levels. ***$P < 0.001$ vs respective control in Student's *t*-test.

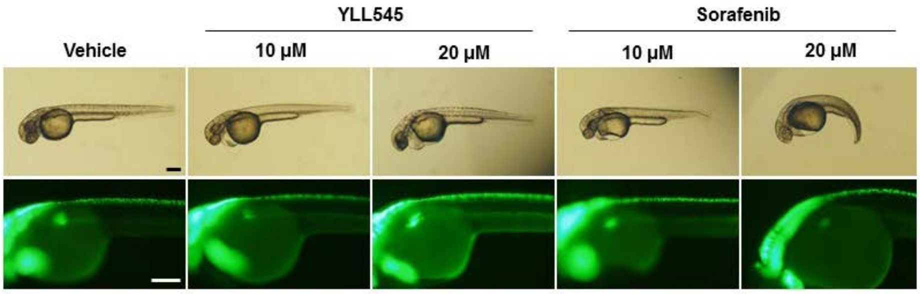

Figure S4. Cytotoxic effect of YLL545 on zebrafish embryonic development. 12-hpf transgenic zebrafish (p48 & cmcl:GFP) embryos were incubated with different concentrations of YLL545 and sorafenib. At 36 hpf, fluorescent assays were performed to visualize the growth of zebrafish embryos. Scale bars, 100 μm.
